# Flood-Rings Production Modulated by River Regulation in Eastern Boreal Canada

**DOI:** 10.3389/fpls.2021.757280

**Published:** 2021-10-28

**Authors:** Alexandre F. Nolin, Jacques C. Tardif, France Conciatori, Yves Bergeron

**Affiliations:** ^1^Institut de Recherche sur les Forêts, Université du Québec en Abitibi-Témiscamingue (UQAT), Rouyn-Noranda, QC, Canada; ^2^Centre for Forest Interdisciplinary Research (C-FIR), Department of Biology/Environmental Studies and Sciences, The University of Winnipeg, Winnipeg, MB, Canada; ^3^Centre d’Étude de la Forêt, Université du Québec à Montréal (UQAM), Montréal, QC, Canada

**Keywords:** dendrohydrology, *Fraxinus nigra* Marsh., earlywood vessels, Ontario (Canada), black ash

## Abstract

In northeastern boreal Canada, the long-term perspective on spring flooding is hampered by the absence of long gage records. Changes in the tree-ring anatomy of periodically flooded trees have allowed the reconstruction of historical floods in unregulated hydrological systems. In regulated rivers, the study of flood rings could recover past flood history, assuming that the effects of hydrological regulation on their production can be understood. This study analyzes the effect of regulation on the flood-ring occurrence (visual intensity and relative frequency) and on ring widths in *Fraxinus nigra* trees growing at five sites distributed along the Driftwood River floodplain. Driftwood River was regulated by a dam in 1917 that was replaced at the same location in 1953. Ring width revealed little, to no evidence, of the impact of river regulation, in contrast to the flood rings. Prior to 1917, high relative frequencies of well-defined flood rings were recorded during known flood years, as indicated by significant correlations with reconstructed spring discharge of the nearby Harricana River. After the construction and the replacement of the dam, relative frequencies of flood rings and their intensities gradually decreased. Flood-ring relative frequencies after 1917, and particularly after 1953, were mostly composed of weakly defined (less distinct) flood rings with some corresponding to known flood years and others likely reflecting dam management. The strength of the correlations with the instrumental Harricana River discharge also gradually decrease starting after 1917. Compared with upper floodplain trees, shoreline trees at each site recorded flood rings less frequently following the construction of the first but especially of the second dam, indicating that water level regulation limited flooding in the floodplains. Compared with the downstream site to the dam, the upstream ones recorded significantly more flood rings in the postdam period, reemphasizing the importance of considering the position of the site along with the river continuum and site conditions in relation to flood exposure. The results demonstrated that sampling trees in multiple riparian stands and along with various hydrological contexts at a far distance of the dams could help disentangle the flooding signal from the dam management signal.

## Introduction

Over the last decades, extreme floods and droughts have become more recurrent and severe in boreal eastern Canada (Buttle et al., 2016; Bush and Lemmen, 2019; Aygün et al., 2020). Understanding if these recent trends are part of the natural hydrological variability is critical and studies remain complicated due to the lack of long instrumental hydrological series (Mortsch et al., 2015; Bush and Lemmen, 2019; Pellerin, 2019). Most gage stations in remote northern rivers were installed in the 1920s following the construction of water regulation systems or hydroelectric facilities, which reduced the availability of natural records (Pellerin, 2019; Nolin et al., 2021b). Therefore, biological proxies such as tree rings have provided hydrological time series in areas that lack instrumental records and have allowed dendrohydrologists to extend existing instrumental records when available.

Tree-ring proxies, among others, have been used to successfully reconstruct monthly, seasonal, and annual discharge (Boucher et al., 2011; Nolin et al., 2021a), lake-level fluctuations (Lemay and Bégin, 2008), droughts (Girardin et al., 2006), and floods (Tardif et al., 1997b; Boucher et al., 2011). Recent studies of anatomical changes in tree rings of periodically flooded trees (i.e., flood rings) have demonstrated their effectiveness in detecting major historical floods and comparing their magnitude (St. George et al., 2002; St. George and Nielsen, 2003; Tardif et al., 2010; Therrell and Bialecki, 2015; Kames et al., 2016; Meko and Therrell, 2020; Nolin et al., 2021a; Tardif et al., 2021a). In riparian ring-porous genus (e.g., *Quercus, Fraxinus*), flooding of tree stems at the time of earlywood formation resulted in decreased earlywood vessel cross-sectional areas (i.e., flood ring; St. George et al., 2002; Copini et al., 2016). Flood rings induced under experimental flooding conditions for amplitudes ranging from 3, 6, or 8 weeks in 4-years-old *Quercus robur* trees indicated that their occurrence was independent of the sapling age and of flooding duration (Copini et al., 2016). Developing quantitative earlywood vessel chronologies led to a significant reconstruction of high spring river discharge compared to the sole use of ring-width chronologies (Nolin et al., 2021a). Indeed, the ring width of riparian trees showed little, and often contrasting, association with flooding (Kames et al., 2016; Tardif et al., 2021b). For example, in Lake Duparquet, the radial growth of black ash (*Fraxinus nigra* Marsh.) in lowland floodplains (Tardif and Bergeron, 1993, Tardif et al., 1997a; Kames et al., 2016; Nolin et al., 2021a; Tardif et al., 2021b) and tamarack (*Larix laricina* K. Koch) in alluvial bogs (Girardin et al., 2001) showed a negative impact of spring flooding, while radial growth of eastern white cedar (*Thuja occidentalis* L.) in upper floodplains showed a positive impact of spring flooding (Denneler et al., 2010). Few consistent responses have been found between tree-ring width and discharge in riparian bur oak (*Quercus macrocarpa* Michx.) along the Red River (Manitoba, Canada; St. George and Nielsen, 2003), but tree-ring widths of riparian European ash, sampled along the Warta River in Poland, demonstrated a positive and significant correlation with previous fall (September to January) and current July and September maximum river flow (Koprowski et al., 2018). The presence and/or absence of flood rings in tree rings of ring-porous trees depends, however, on their exposure to spring flooding. Depending on the hydrological context, flood rings varied in relative frequency and intensity (Nolin et al., 2021b; Tardif et al., 2021b). For instance, Tardif et al. (2021a) and Tardif et al. (2021b) demonstrated that flood rings were formed mainly in *F. nigra* trees located in the lower floodplain but not in trees exposed to the same hydrological regime, but growing at a higher elevation. Fewer trees recorded flood rings in floodplains of regulated rivers compared with those of natural rivers, and flood rings in regulated rivers were also less distinct (intense) than those in natural rivers (Nolin et al., 2021b). Compared to the hydrological records from natural rivers, the natural discharge variability is masked by dam regulation and management maneuvers in regulated-river hydrological records (Déry et al., 2016) with a particular reduction in maximum daily and annual peak discharge downstream of dams (Williams and Wolman, 1984; Magilligan and Nislow, 2005; Graf, 2006). After water-level regulation, floodplain trees would be inundated less frequently due to flood attenuation by the dam. The tree rings would therefore be less likely to record all the same years of flooding as in nearby natural rivers. The impact of dam regulation on riparian forests could thus alter their ability to provide proxy (natural) hydrological data when trees have coped with altered hydrological regimes.

River regulation and dam management alter water availability for both upstream and downstream riparian forests in space and time and are further influenced by an interplay of factors such as plasticity of species, soil moisture, microtopography, etc. (Nilsson and Berggren, 2000; Magilligan and Nislow, 2005; Stella and Bendix, 2019). Downstream of a dam, the water table may be lowered, and the flood peaks attenuated with the frequency and magnitude of flooding reduced, suppressed, or shifted in time depending on reservoir management (Ashmore and Church, 2001; Nilsson and Berggren, 2000; Magilligan and Nislow, 2005; Graf, 2006). The composition of floodplain forests downstream of a dam often shifts to more drought-tolerant species than in the predam period with a loss of old-growth trees (DeWine and Cooper, 2007; Stallins et al., 2010; Schook et al., 2016). The response time of riparian forests varies widely across hydrological contexts. For example, Smith et al. (2013) found that the regulation of the Apalachicola River, initiated in the 1970s, reduced flood disturbance that positively affected tree age, and negatively affected radial growth and recruitment.

Hydrological effects upstream of dams have been less commonly studied (Petts, 1980; Evans et al., 2007; Baena-Escudero et al., 2021). In the short term, water regulation raises the water level for riparian forests upstream of a dam in the portion of the river under the influence of the dam (reservoir) resulting in forest mortality or composition change (Nilsson and Berggren, 2000), or in the loss of trees/species presenting flood rings. In the longer term, a dam can also cause progressive siltation reducing the river cross-section upstream of the dam. For example, Baena-Escudero et al. (2021) demonstrated that the siltation induced by dams on the Guadalquivir River in Spain, laden with silty sand sediments, resulted in a reduction in the discharge capacity of the river during floods less than 100 years after the dams were built. As a result, higher water levels were reached after siltation, for an equivalent discharge before siltation, increasing the frequency and duration of flooding of the floodplains along the reservoir (Baena-Escudero et al., 2021). Slater and Villarini (2016) also noted that trends in flood frequencies among American rivers were different for some river basins that had undergone regulation or reduction in the capacity of the main river channel (urbanization). However, the length of the dam reservoir varies depending on the characteristics of the river and of the dam (Nilsson and Berggren, 2000). Further upstream of this reservoir, riparian forests, and flood dynamics are not affected and retain their natural attributes.

The impact of river regulation on radial tree growth has been little studied, but dendrochronological studies suggest that river damming generally has an adverse effect on the radial growth of riparian trees. Downstream of dams, regulation increases the sensitivity of riparian tree growth to drought and low water levels (Reily and Johnson, 1982; Salzer et al., 1996; Coble and Kolb, 2012). As a result, tree growth is more highly correlated to discharge during the predam period and to drought (low discharge) and precipitation during the postdam period (Reily and Johnson, 1982; Stromberg and Patten, 1990; Salzer et al., 1996; Coble and Kolb, 2012; Netsvetov et al., 2019). The damming also tends to reduce the ring-width variability in the postdam period (Salzer et al., 1996). Reily and Johnson (1982) compared regulation effects on radial tree growth of several riparian species along elevation gradients in the Missouri River and demonstrated that tree growth downstream of an altered hydrological regime responds in variable ways depending on, among others, tree species and elevation to water levels.

The aim of this study was to determine how discharge regulation by dams affects both ring width and the production of flood rings along with various regulated hydrological contexts. More precisely, we asked how are radial growth and flood-ring production of riparian *F. nigra* trees are impacted by the construction of hydrological dams, and if the ability of trees to record high-magnitude flood rings differ according to distance to the shoreline and among different sites located upstream and downstream of the dam. It was hypothesized (i) that flood rings would present no differences between sites prior to dam construction whereas (ii) after dam construction, flood rings would be recorded more abundantly and be of higher intensity upstream. It was also hypothesized that dam construction would reduce ring width and its variability, as well as alter the correlation structure with discharge and precipitation. In addition to enhancing our understanding of the impact of dam regulation on the ability of riparian *F. nigra* trees to record hydrological events, this study addresses sampling strategies that maximize the success of generating flood records of riparian trees in a context of hydrological regulation.

## Materials and Methods

### Study Area

**FIGURE 1:**
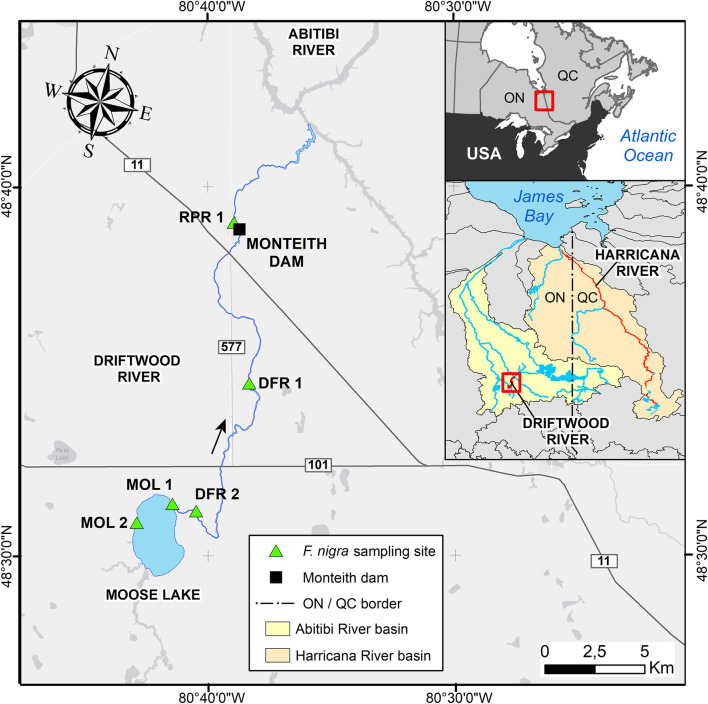
Map of the study area. The geographic location of the Driftwood River at the border between Ontario and Québec in Canada (upper right inset) and within the Abitibi River basin (lower right inset). Locations of the *Fraxinus nigra* sampling sites along the Driftwood River are coded as follows: MOL - Moose Lake; DFR - Driftwood River; RPR - Rapid River.

**FIGURE 2:**
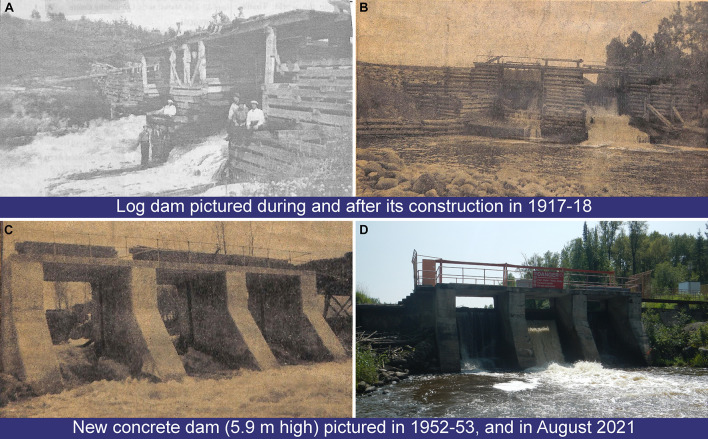
The old and the new on Driftwood River at Monteith. First log dam **(A,B)** and second concrete dam **(C)** in the years of their construction as compared to today structure **(D)**. Historical photos **(B,C)** by Mrs. C. Clifford were published in an unknown local newspaper on May 6, 1953. All historical photos retrieved from the digitalized archives of the Monteith Women Institute Tweedsmuir Community History, a courtesy of the Federated Women’s Institutes of Ontario (FWIO; https://collections.fwio.on.ca). Photo in August 2021 by AFN.

**FIGURE 3:**
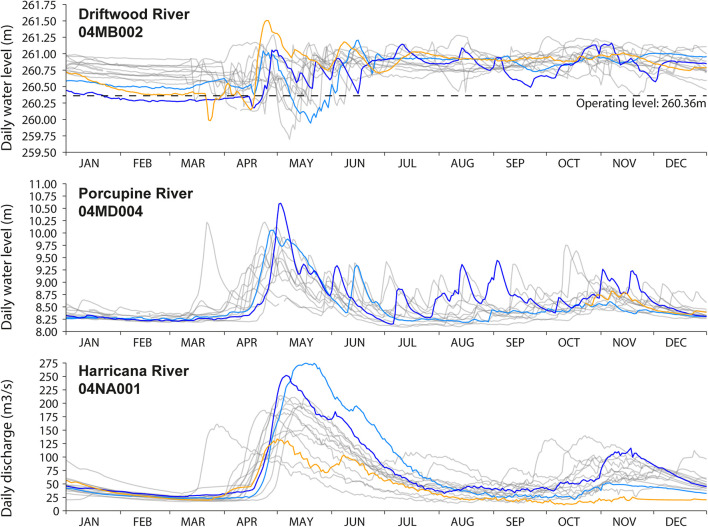
Comparison of the daily water level of the Driftwood River downstream of the Monteith dam (2006–2020) with the daily water levels of the unregulated Porcupine River at Hoyle (∼50 km west; 2007–2019) and the daily discharge of the unregulated Harricana River at Amos (∼200 km east; 2006–2020). Daily data for the year 2007 are incomplete at the Porcupine River gage station. Data were extracted from the historical hydrological station data of the Water Survey Canada. The years of maximum discharge or water level found during the 2006–2020 period and in the three rivers are highlighted in color. The light blue line represents the year 2019 (maximum discharge in the Harricana River), the dark blue line represents the year 2013 (maximum water level found in the Porcupine River), and the orange line represents the year 2007 (maximum level found in the Driftwood River). The dashed line in the upper panel depict the operating level (260.36 m) of the Monteith dam.

The study area is located on the Driftwood River, meandering in the clayey and peatland plains of northeastern Ontario at the southern fringe of the boreal forest (Figure 1). The source of Driftwood River is located near Lipsett Lake (48°19′49″ N; 80°42′46″ W). After flowing north to reach Moose Lake, the river ends in the Abitibi River (Figure 1). Because the head of the Driftwood River basin is not easily accessible, the sampling started on the shores of Moose Lake (11 km^2^) to the confluence with the Abitibi River (Figure 1). The Driftwood River was flowing naturally until the construction of a log dam in 1917–1918 at Monteith (Ontario Ministry of Natural Resources and Forestry [OMNRF], 2019; Figures 1, 2). The height of the log dam is not known but the structure was built with two spillways to store the water in a reservoir and to supply electricity (60-cycle power) to a Demonstration Farm for the Ontario Government. In 1941, the generator was downsized to a 25-cycle electric power to light a prisoners-of-war camp. The reservoir and spillways were maintained but apparently abandoned. From 1952 to April 1953, the old log dam was replaced at the same location by a 5.9-meter-high and 52-meter-long concrete gravity structure to supply water for an industrial farm (The old and the new on Driftwood River, 1953). It is possible that the dam was built on an existing rapid or on a natural constriction of the riverbed as is the case for other rivers in the region. Monteith dam is owned and operated by the Ontario Ministry of Natural Resources to control the Driftwood River as far upstream as Moose Lake. Most of the year, the dam is maintained at a fixed operating level of 260.36 m (Figure 3; Ontario Ministry of Natural Resources [OMNR], 2004). The dam is operated solely at spring for flood mitigation with manually operated stop logs to maintain Moose Lake level at, or below, 260.66 m (Figure 3; Ontario Ministry of Natural Resources [OMNR], 2004).

Upstream of the Monteith dam, a gage station (04MB002; Water Survey of Canada, 2021; Figures 1, 3) has been in service since 2006 to record water levels about 10 m upstream of the dam. While of short duration, the daily hydrographs indicate that the Driftwood River follows a boreal regime with a peak response to ice and snowmelt in spring lasting for several weeks. Relatively high water levels are maintained through the summer, probably because of the dam, and with a late recession in winter a few months before the freshet (Figure 3). There is no record of the Driftwood River hydrological regime prior to the dam, making it impossible to assess the exact impact of the dam on flood magnitude or mean water levels upstream and/or downstream. The nearest unregulated hydrological record is for the Porcupine River (04MD004), located about 50 km to the west with data from 2007 to 2019. The drainage areas of the gage stations on the Driftwood and Porcupine Rivers are similar (537 km^2^ compared to 408 km^2^) allowing a direct comparison. The nearest, longest unregulated records are for the Harricana River (04NA001 and 04NA002), located about 200 km to the East, with data from 1915 to 2021, over a larger gross drainage area (3, 680 km^2^).

### Tree-Ring Data

Tree-ring data were collected in the summer of 2017 to investigate the spatial coherency of spring floods in eastern boreal Canada (Nolin et al., 2021b). A total of 43 living *F. nigra* trees were sampled in five sites representing different hydrological contexts with site Rapid River (RPR) 1 located 1 km downstream of Monteith dam; site Driftwood River (DFR) 1, located 10 km upstream of the dam; site DFR 2 located 25 km upstream on the tip of a meander and 1 km downstream of the estuary of Moose Lake (MOL); site MOL 1 located 27 km upstream at the estuary of Moose Lake; and site MOL 2 located 31 km upstream on the opposite shore of Moose Lake estuary (Figure 1 and Table 1). *Fraxinus nigra* trees growing on the shoreline of the Driftwood River are situated close to their northern distribution limit. The species grows on fine waterlogged clay soils and occurs in pure or mixed stands in association with eastern white cedar and balsam poplar (*Populus balsamifera* L.; Tardif and Bergeron, 1992; Tardif and Bergeron, 1999; Denneler et al., 2008). The species is tolerant to water-level fluctuations (Sims et al., 1990) and was sampled in the lower floodplains where sensitive fern (*Onoclea sensibilis* L.) is indicative of long-lasting floods (Tardif and Bergeron, 1992). Dominant trees were selected, and two cores were collected on trees with the largest diameter with the aim to produce long chronologies. Coring was done with a 5 mm increment borer in two opposite directions to reach the tree center and as close to the tree base as possible. Diameter at breast height was recorded as well as the distance of each tree to the shoreline. Each core sample was prepared according to standard dendrochronological procedures (Phipps, 1985) and sanded with a progressively finer grit. Prior to cross-dating, each sample was inspected for the presence of flood rings by two observers, each processing half of the samples. In *F. nigra*, flood rings are characterized by a noticeable increase in earlywood vessel number which is accompanied by a noticeable decrease of earlywood vessels area (Tardif et al., 2010; Kames et al., 2016; Nolin et al., 2021a,b; Tardif et al., 2021a). A dual numerical code was used to record the level of distinctiveness of flood rings with an F1 code indicating a weakly defined flood ring and an F2 code indicating a well-defined flood ring (Nolin et al., 2021b; Tardif et al., 2021a). Cross-dating was then performed using the previously defined regional *F. nigra* pointers years (Tardif et al., 1997a; Kames et al., 2016; Nolin et al., 2021b). The age of each tree was determined from the oldest core sample and was either the age of the pith or the age of the last visible tree ring when the pith was not present on the core. Ring widths were measured with CooRecorder (v9.0.1; Larsson, 2003a) on 2400 dpi scanned images and were validated with CDendro (v9.0.1; Larsson, 2003b) and COFECHA (Holmes, 1983).

**TABLE 1:** Location of sampling sites.

Site	No. trees	Position to dam	Latitude/Longitude	Period
RPR 1	8	1 km downstream	48°38′53″N; 80°40′42″W	1930–2017
DFR 1	6	10 km upstream	48°34′31″N; 80°40′05″W	1917–2017
DFR 2	10	25 km upstream	48°31′03″N; 80°42′15″W	1879–2017
MOL 1	8	27 km upstream	48°31′15″N; 80°43′13″W	1898–2017
MOL 2	11	31 km upstream	48°30′44″N; 80°44′39″W	1852–2017

*Data are ordered by increasing the distance to the dam from top to bottom. A period is the length of flood-ring records.*

### Statistical Analysis and Independent Data

Both, flood-ring relative frequencies and ring-width chronologies, were generated. Flood-ring series were produced from visual identification of flood rings and of their intensity (F1 and F2) by two expert observers. Tardif et al. (2021a) demonstrated that both flood rings and their intensity could be identified with high consistency by novice and expert observers and that visual identification of flood rings could provide accurate and reproducible semiquantitative data. Flood-ring series were pooled by the tree, keeping the code with the maximum intensity for each year (0 < F1 < F2; Nolin et al., 2021b). Flood-ring categories have been shown to respond to the same hydroclimatic variables and to be complementary with F1 observed in most years, F2 observed in known high-flood years, and both F1 and F2 being almost absent from unflooded control sites (Nolin et al., 2021b; Tardif et al., 2021a). The relative frequencies of F1 and F2 by sites were then calculated by dividing their respective sum by the number of trees per year. Both F1 and F2 relative frequencies were also summed (F12). Ring-width measurement series were averaged by the tree (*n* = 2 cores) and the old non-overlapping portion was kept (*n* = 1 core) to maximize temporal coverage. Ring-width series were standardized by dividing each tree series by its mean to conserve the integrity of the low-frequency variations (Cook and Kairiukstis, 1990). Standardized ring-width series and F1, F2, and F12 relative frequencies were then averaged by sites. To assess a possible effect of distance from the shoreline, each of those series was grouped into two distance classes representing shoreline trees [0–10 m] and floodplain trees [10–100 m].

Non-parametric change point analysis was performed on mean site ring-width chronologies using the “ecp” package (v3.1.3; James and Matteson, 2014). A univariate change point analysis was performed for each site, and multivariate analysis was performed using all mean site chronologies at once. Multivariate analysis of change points was performed by successive common periods, removing the youngest chronology each time until there were only two chronologies left. Change points were estimated by iterative hierarchical segmentation of chronologies and tested for significance using 1,000 Monte Carlo permutation tests (Matteson and James, 2014).

Flood-ring relative frequencies and ring-width chronologies were finally compared to four independent instrumental and proxy series. First, daily discharge data from the unregulated Harricana River (1915–2021; Water Survey of Canada, 2021) were used (Figure 1). Second, both a reconstruction of the spring (April 15-June 30) discharge and a chronology of flood-ring relative frequency for the Harricana River were available for the period 1771–2016 (Nolin et al., 2021a,b). The reconstructed Harricana River discharge data are highly correlated with the instrumental data (*r* = 0.819, *p* < 0.001, 1915–2016) and can be used as a proxy for the period before 1915 (Nolin et al., 2021a). At last, precipitation data were also retrieved from gridded CRU TS4.04 (Harris et al., 2020) using the KNMI Climate Explorer^1^ (Trouet and Van Oldenborgh, 2013). Spring (March-April-May) precipitation data were averaged over the 0.5° × 0.5° grid cells corresponding to the spatial extent of the sampling sites (48°N; −80°E to 48.5°N; −80.5°E; Figure 1) and covering 1901–2019.

The flood-ring relative frequencies (F1, F2, F12) were compared among sites using bootstrapped Spearman correlation coefficients (Legendre and Legendre, 2012). Each correlation was calculated on 10,000 random samplings of the chronologies to approximate the sampling distribution of the Spearman correlation coefficient and to return its mean (Efron, 1992), using the “boot” package (Canty and Ripley, 2021). Ring-width chronologies were compared among sites and with the aforementioned hydroclimatic data using 40-year moving windows of bootstrapped Pearson correlation coefficients lagged backward by 5 years (Legendre and Legendre, 2012) using the “treeclim” package (Zang and Biondi, 2015). All statistical analyses were conducted in the R statistical environment (R Core Team, 2021).

## Results and Discussion

### Impact of Regulation on Stand Structure

The comparison of daily hydrographs of regulated and unregulated rivers illustrates the reduction of spring flood peaks and the maintenance of high-water levels throughout the year in the Driftwood River compared to a natural regime (Figure 3). Comparing the years of the highest water levels and discharge in the Driftwood, Porcupine, and Harricana Rivers also shows that years of high-water levels in the regulated Driftwood River do not necessarily correspond to years of high-water levels or high discharge in the two other natural rivers (2007 orange line in Figure 3). In the natural Porcupine and Harricana Rivers, the years 2013 and 2019 are known as major flood years and show comparable high-water levels and discharge (blue lines in Figure 3).

**FIGURE 4:**
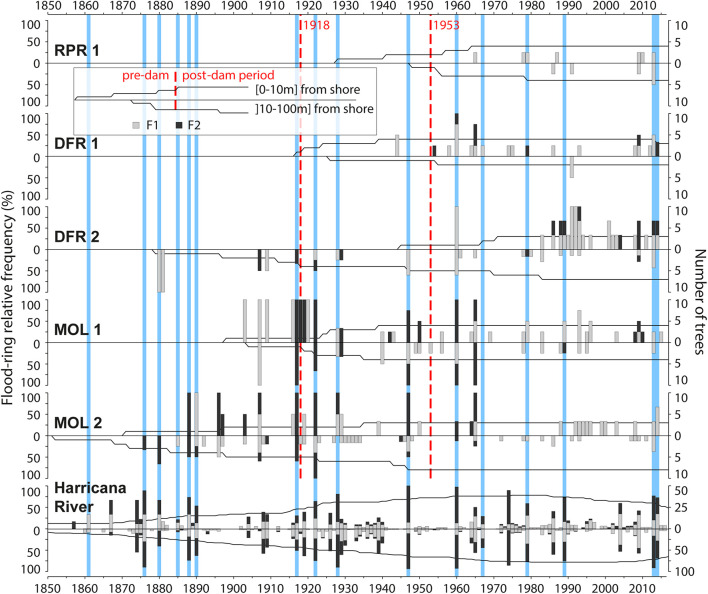
Flood-ring chronologies for each of the five sites and organized by distance classes to the shoreline (0–10 and 10–100 m) and for the naturally flowing Harricana River. Trees close to the shoreline (0–10 m) are shown with positive bars and trees from the floodplain (10–100 m) with negative bars. Gray and black histograms are, respectively, F1 and F2 flood-ring relative frequencies, and the black solid line in the background indicates temporal replication among the trees sampled per year. Vertical red dashed lines indicate dates of construction of the first and second Monteith dam. Blue vertical bars indicate the years with the highest reconstructed spring discharge for the Harricana River (threshold value = 151.3 m^3^/s; Nolin et al., 2021a,b).

The ages of the trees sampled on the Driftwood River ranged from 32 to 166 years with differences observed from upstream to downstream (Table 2). The oldest and largest *F. nigra* trees were located at the greatest distance upstream of Monteith dam (MOL 2) while the youngest and smallest trees were found at the site right downstream of the dam (RPR 1) and at the closest site upstream of the dam (DFR 1; Table 2 and Figure 4). Site DFR 2 presented the most dispersed age class compared with others with tree ages ranging from 38 to 138 years (Table 2 and Figure 4). The difference in age between upstream and downstream sites was consistent, although the maximum age of a few trees was underestimated because of rotten parts (14 of 43 trees) and could therefore be assumed to be older. The maximum ages of the trees suggest that sites DFR1, DFR2, MOL1, and MOL2 were present prior to the construction of the log dam in 1917–1918 and that RPR1 was established prior to the construction of the second dam in 1952–1953 (Table 2 and Figure 4). On the shores of Moose Lake (MOL 1, MOL 2), *F. nigra* trees formed dense and thick fringe forests from the shoreline to a maximum of 100 m in the floodplain. Further downstream on the Driftwood River (DFR 2, DFR 1, RPR 1), *F. nigra* trees were organized in narrow linear stands along the river and extended to a maximum of 30 m in the floodplain. The old age and extent of the stands suggest that *F. nigra* trees at Moose Lake have survived the dam constructions. In the nearby Lake Abitibi, the first damming of the lake in 1915 raised the water level of ca. 1.2 m and caused the mortality of the riparian fringe forest (Denneler et al., 2008). Investigating the spatial coherency of flood rings, Nolin et al. (2021b) found significatively shorter flood-ring records in regulated rivers compared with natural rivers, which suggested that regulation may have permanently eliminated old-growth floodplain forests. Several studies also reported a negative effect of peak flow regulation recruitment and establishment of riparian trees downstream of a dam (DeWine and Cooper, 2007; Smith et al., 2013; Schook et al., 2016). In this study, no additional stands of *F. nigra* were found downstream of RPR 1. Besides an effect of regulation, other factors could also explain the age difference between sites (oldest upstream, youngest downstream) such as land-use changes, timber harvest, or natural dynamic of seeds propagation. However, no evidence of land-use changes and lowland forest harvesting was found in the archives consulted (Geography of Monteith, n.d.; The old and the new on Driftwood River, 1953). Seeds of *Fraxinus* trees (samaras) are morphologically adapted to be dispersed by wind, and dispersal by water current in periodically flooded riverine lowlands has also been reported (Merritt and Wohl, 2002; Schmiedel and Tackenberg, 2013). *F. nigra* sexual regeneration at Lake Duparquet was also positively influenced by high water levels during spring and early summer, while the species also regenerate by vegetative reproduction (Tardif et al., 1994). In the Saône River in France, flooding favored *Populus* regeneration by eliminating seedlings of other flood-intolerant species (Astrade and Bégin, 1997). It can thus be hypothesized that river regulation may result in a regeneration deficit of riparian *F. nigra* downstream of the dam and upstream at the reservoir. Since only old trees were sampled in this study, the data do not provide evidence of an effect of Monteith dams on *F. nigra* recruitment in the Driftwood River floodplains. A future research avenue should thus investigate age classes and seedling perennity among floodplain forests and between hydrological contexts.

**TABLE 2:** Characteristics of *Fraxinus nigra* stands.

Site	Diameter	Age	Distance to shore
RPR 1	27.5 ± 6.3	68.9 ± 15.3	13.4 ± 11.8
DFR 1	26.7 ± 5.8	89.3 ± 10.2	6.8 ± 6.4
DFR 2	35.4 ± 10.9	85.0 ± 31.2	35.9 ± 18.0
MOL 1	33.9 ± 7.1	98.4 ± 14.9	8.8 ± 5.5
MOL 2	48.5 ± 9.9	122.1 ± 31.3	29.5 ± 25.6

*Mean values and standard deviation by site indicating (1) stem diameters at breast height, (2) maximum tree age, and (3) their position in the floodplain relative to the shoreline.*

*Data are ordered by increasing distance to the dam from top to bottom.*

### Flood-Ring Relative Frequencies (Predam Period)

The flood-ring chronologies covered 1852–2017 with a common overlap period between 1930 and 2017 (Table 1). Little flood-ring data were available prior to the construction of the first log dam (1917–1918; Figure 4). The most consistent flood-ring record for the predam period was found at the most ancient and upstream site MOL 2 (Figure 4). The high spring discharge years evidenced in the F12 relative frequencies from the Harricana River were identified by the years 1880, 1888, 1890, and 1917 recorded by 33 to 100% of the trees growing on the shoreline and in the floodplain of Moose Lake with mainly F2 flood rings (Figure 4). F12 relative frequencies from MOL 2, MOL 1, and DFR 2 also correlated positively (*r* = 0.25–0.50) and significantly (*p* < 0.05) with the reconstructed Harricana River spring discharge (Table 3). Few years exhibited high F1 and F2 relative frequencies while not being equaled in the Harricana River records and supported the regional contrasts evidenced in years 1896 and 1907 by Nolin et al. (2021a, b). Those 2 years were similarly characterized by high spring discharge and mid to high frequency of flood rings at Lake Duparquet (20 and 81%, respectively) and in the Little Abitibi River (38 and 76%, respectively; Nolin et al., 2021a,b). Other years with high F12 relative frequencies for this period in the Driftwood River (DFR1, DFR2) demonstrated a good agreement with MOL 2 and with the reconstructed Harricana River spring discharge (Figure 4 and Table 3). Of importance is that the relative frequencies of F1 and F2 were higher in trees growing on the shoreline than in the floodplain, where more abundant F1 with lower relative frequencies were found (Figure 4). Not all years with F2 values in shoreline trees were matched by comparable F2 values in floodplain trees, as is the case for the Harricana River flood-ring record (Figure 4). Tardif et al. (2021, 2021b) showed that flood rings gradually disappeared from low to high elevation floodplains to non-flooded upland stands and that microtopography led to differences in exposure of trees to flooding. This result suggests that natural historical floods may have flooded for short distances to the shoreline, which would further increase the sensitivity of the *F. nigra* forests to a change in flood regime or water levels by dams. Overall, the results from the predam period compared well with the findings from other regulated rivers in the area that showed a high spatial coherency with most flood rings recorded in natural rivers prior to the period of dam implementations in the region (1920–1930s; Nolin et al., 2021b).

**TABLE 3:** Comparison of F12 flood-ring relative frequencies with instrumental and reconstructed mean spring (April 15–June 30) discharge of the naturally flowing Harricana River and by distance to the shoreline (0–10 and 10–100 m).

	Pre-dam period (*)				After the first dam: 1918–2016				In-between the two dams: 1918–1952				After the second dam: 1953–2016			
	[0–10 m]		[10–100 m]		[0–10 m]		[10—100 m]		[0–10 m]		[10–100 m]		[0–10 m]		[10–100 m]	
	*rho*	*p*	*rho*	*p*	*rho*	*p*	*rho*	*p*	*rho*	*p*	*rho*	*p*	*rho*	*p*	*rho*	*p*
RPR 1	ND	ND	ND	ND	ND	ND	ND	ND	ND	ND	ND	ND	0.04	0.742	0.23	0.075
DFR 1	ND	ND	ND	ND	**0.23**	0.024	ND	ND	−0.23	0.204	ND	ND	**0.41**	0.001	−0.05	0.674
DFR 2	ND	ND	**0.35**	0.005	ND	ND	**0.27**	0.008	ND	ND	**0.49**	0.006	**0.28**	0.034	0.18	0.16–0
MOL 1	**0.31**	0.013	**0.25**	0.051	**0.27**	0.007	**0.32**	0.002	0.26	0.172	**0.48**	0.008	**0.27**	0.041	0.20	0.120
MOL 2	**0.38**	0.003	**0.50**	0.000	**0.28**	0.007	0.15	0.152	**0.60**	0.000	0.05	0.721	0.11	0.427	0.20	0.131

*Spearman correlation coefficients (*rho*) are given after calculating 10,000 bootstrap iterations.*

*Bold characters denote significant *rho* coefficients at *p* < 0.05.*

*Spearman coefficients were calculated for four periods (1) The predam period before the first log dam (1850–1917), (2) After the first log dam (1918–2016); (3) In between the first log dam and the second concrete dam (1918–1952); and (4) After the second concrete dam (1953–2016).*

(*) Note that for the predam period, the number of years varies among sites and distance classes with DFR 2 (1879–1917), MOL 1 (1898–1917), MOL 2 (1852–1917; Figure 4 and Table 1).

*The predam period has been calculated with the reconstructed Harricana River discharge, the other periods with the instrumental Harricana River discharge.*

*ND: no data.*

### Flood-Ring Relative Frequencies (Postdam Period)

In contrast to the flood-rings chronology from the unregulated Harricana River, those from the Driftwood River tracked the hydrological changes that occurred after the implementation (1917) and the replacement (1953) of the Monteith dam. The most striking features were the decrease in the flood-ring relative frequencies and the decrease in the number of F2 in the postdam period (Figure 4). This response was also seen in the decrease in the number of sites with a significant correlation of F12 relative frequencies with the mean Harricana River instrumental spring discharge (Table 3).

During the period between the first and the second dam (1918–1952), high F12 relative frequencies with high values of F2 were still recorded during the known high spring discharge years of 1922, 1928, and 1947 at upstream sites MOL 2, MOL 1, and DFR 2 (Figure 4). The F12 relative frequencies from those sites still correlated positively and significantly with the instrumental Harricana River mean spring discharge. However, the correlations from shoreline trees at MOL 1 presented a non-significant (*p* < 0.05) correlation while in a range of the correlation strength found in the other periods (Table 3). Few high values of F12 relative frequencies were observed in floodplain trees growing on the MOL 2 site compared to the predam period. Abundant F1 flood rings of low relative frequency (<20%) were also observed in floodplain trees at the MOL 2 site during the 1917–1952 period, but not in shoreline trees, which contrasts with the other flood-ring records from the other four sites (Figure 4). Like the predam period, higher F12 relative frequencies, and mostly F2 flood rings, were found for trees close to the shoreline compared to floodplain trees (Figure 4).

After the replacement of the dam in 1953, values of F12 relative frequencies decreased at all sites compared with the previous two periods, with mainly F1 being recorded. The lowest F12 relative frequencies were found near the dam (RPR 1 and DFR 1) and the highest values were found upstream and at a far distance from the dam (MOL 2, MOL 1, DFR 2). Site RPR 1 showed almost no flood rings (37.5% at maximum in three of eight trees) although the age of the stand does not allow comparison with a predam period. Experimentally induced flood rings, however, have been shown to form in 4-year-old pedunculate oak (*Q. robur* L.) trees suggesting that young riparian forests should be able to record flood history (Copini et al., 2016). The absence of flood rings at RPR 1 might thus reflect the river regulation.

At the other sites, flood rings were primarily F1, with high F2 relative frequencies found in both known regional flood years and in random years. High spring discharge years 1960, 1967, 1979, 1989, 2013, and 2014 were evidenced in the Harricana River record (Figure 4). The 1960 flood was reconstructed as the highest discharge of the last 250 years in the Harricana River (Nolin et al., 2021a) and showed very high F12 relative frequencies (approaching 100%) in multiple natural regional rivers (Nolin et al., 2021b). In the Driftwood River, high flood-ring relative frequencies of F1 and F2 types were still found in 1960 after the dam was replaced (Figure 3). Other years with high F12 relative frequencies such as 1965 or 2007 may result from the dam management in the Driftwood River, or from regional contrasts in spring flooding (Figure 4). For instance, the year 1965 was characterized by high ice-scar frequency in Lake Duparquet (Tardif et al., 1997b) and by mid to high F12 flood-ring relative frequencies in Lake Duparquet (69%) and regional natural rivers (44%; Nolin et al., 2021b). The year 2007 was the highest spring water level recorded in the Driftwood River upstream of the Monteith dam and over the period 2006–2020, which was not the case in the natural Harricana River (Figure 3). The mean spring discharge of the Harricana River for the year 2007 (91.5 m^3^/s) was well below the discharge threshold used to define high spring discharge years in this river (151.3 m^3^/s, Nolin et al., 2021a,b).

Floodplain trees recorded very low F12 relative frequencies compared with the shoreline trees at all sites (Figure 4). No significant correlation with instrumental mean Harricana River spring discharge was either found for F12 from floodplains trees at all sites (Table 3). Trees of site MOL 2 almost stopped recording flood rings after 1960 and it can be assumed that the decrease in the flood peak regime induced by the dam has ended the flooding of this site while maintaining the old-growth forest. The F12 records from shoreline trees at other sites upstream of the dam (MOL 1, DFR 2, DFR 1) still correlated positively and significantly with the instrumental Harricana River mean spring discharge (Table 3). Higher F12 relative frequencies were found after ∼1980 in the shoreline trees at site DFR 2, which may indicate an intensification of spring water storage by the dam over this period compared to the other sites over the same period, and to the period before 1980 at site DFR 2 (Figure 4).

Throughout the flood-ring records in the postdam period, no extremely high relative frequencies were found during the known date of town settlement, or road and bridge construction. The small village of Driftwood city established on the east side of the Driftwood River was destroyed in the 1916 fire. The first buildings of the actual Monteith were built in 1916, after the fire, and on the opposite side of the river (Geography of Monteith, n.d.). No particularly high flood-ring relative frequencies were neither found for the years of the construction or modification of roads and bridges. The Ontario Highway 11 and Secondary Highway 577 (Figure 1) were built in 1920 and 1956, respectively, and were modified in 1953, 1958, and 1959 (Highway 11) and in 1978 (Highway 577) sometimes associated with muskeg excavations.^2^ While the number of samples used in this study is a limiting factor, the overall findings agree with those from regional regulated rivers where trees recorded few flood rings and of low intensity (F1) after the period of dam constructions in the region (1920–1930s; Nolin et al., 2021b).

The exclusion of low F12 frequencies or partitioning of F12 frequencies between F1 and F2 flood rings may allow separating years of a major flood from years of high-water stage induced by dam management or other anthropogenic alterations (e.g., bridge construction, muskeg excavations). This may become important in a context where it is not possible to compare flood-ring relative frequencies to regional hydrologic records (instrumental or reconstructed). The high F12 relative frequencies that are present in both, shoreline trees (F12 > 75%) and floodplain trees (F12 > 50%), indicate that the spring discharge from 1960 to 1965 flooded the shoreline and the floodplain trees after the Monteith dam construction. The years for which F2 have been recorded in both, shoreline and floodplain trees, also indicated that the years 1960, 1965, and the year 2009 inundated the distant floodplain. Different flood-ring responses to those flood years at sites MOL 1 (high relative frequencies) and MOL 2 (low relative frequencies; Figure 4) emphasized that sampling sites with different elevation and flood exposure gradients might help in disentangling major flood years from water-level management events. Moreover, in this study, cores were taken at varying heights due to varying wood quality (i.e., rot, frost scars). It would be interesting to include sampling height in future analyses since flood rings have been shown to solely occur below water level (Copini et al., 2016) and/or to weaken along with stem height and with increasing distance to the ground (St. George et al., 2002). Studying the flood-ring distribution in relation to stem height could provide a proxy for flood height and it would add to the information that can be provided by flood-ring intensity. For instance, Tardif et al. (2021a) noted that flooded *F. nigra* trees in close proximity could record flood rings of different intensity (F1, F2) supporting the idea that sampling height and tree elevation might affect the distinctiveness of flood-ring signature. The authors also noted that the identification error between two observers was greater for weakly defined flood rings (F1) than for well-defined flood rings (F2).

### Ring-Width Chronologies

**FIGURE 5:**
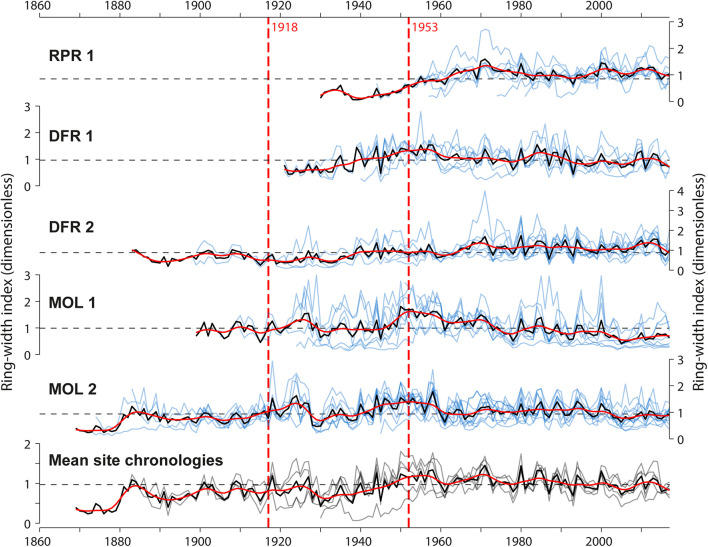
Standardized tree-ring width series (pale blue line) and mean site chronologies (dark black lines), and comparison of mean site chronologies among the five sites. Vertical red dashed lines indicate dates of construction of the first and second Monteith dam. The red curves are 10-year spline functions and depict decadal variations in the mean radial tree growth at each site.

Over the 1930–2017 common period, the mean correlation between the five site chronologies was generally high (*r* = 0.49 ± 0.24 sd) except for the site RPR1, which was weakly correlated with the other sites (*r* = 0.19 ± 0.28 sd). The highest significant correlation among ring-width chronologies was found between the most upstream sites MOL 1 and MOL 2 (*r* = 0.69, *p* < 0.001). The univariate change point analysis identified no change in mean and/or variance in each mean site ring-width chronologies that could be related to the first or second dam construction (Figure 5 and Supplementary Table 1). Significant change points were detected with low consistency in multivariate analysis in years 1909, 1914, 1917, 1941, 1945, 1974, and 1975 (*p* < 0.05) over most of the common periods used for the multivariate change point analysis (Supplementary Table 1). The most significant change point was the year 1974, identified in three different combinations of mean site chronologies (Supplementary Table 1). This year does not correspond to any of the years of the village (1916), road, bridge (1920, 1953, 1956, 1958, 1959, 1978), or dam (1917, 1953) constructions that may have possibly affected the hydrology of the Driftwood River. Grouping the mean site chronologies into distance classes (shoreline and floodplain trees) resulted in no improvement of the results (results not shown). At each site, ring-width chronologies showed little association with instrumental Harricana River mean spring discharge or average spring precipitation for all periods considered (before and after 1953, common period 1930–2016). For example, considering the common period 1930–2016, correlations between ring-width chronologies and, respectively, river mean spring discharge or average spring precipitation were close to zero (mean *r* = −0.02 ± 0.07; mean *r* = −0.07 ± 0.07; *n* = 5).

**FIGURE 6:**
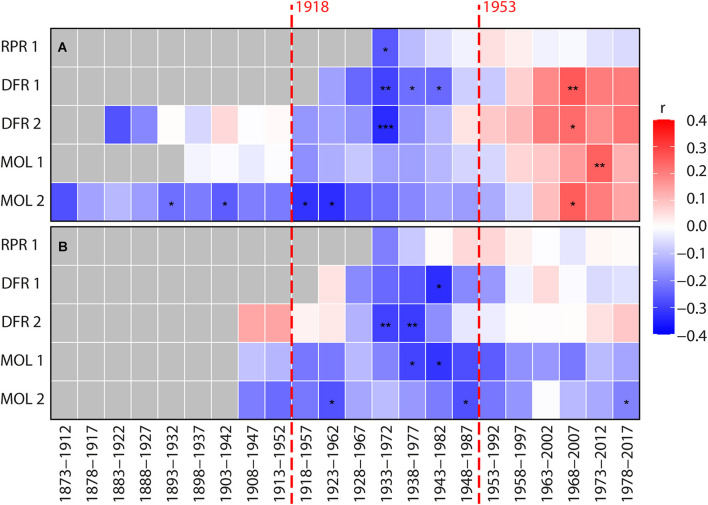
Bootstrapped moving Pearson correlations between individual site ring-width chronologies **(A)** Harricana River spring (April 15–June 30; 1869–2017) discharge, and **(B)** the CRU TS 4.04 mean spring precipitation (MAR-APR-MAY-JUN; 1901–2017). The mean spring discharge of the Harricana River used in this analysis is constructed from the reconstructed (1869–1914) and instrumental (1915–2017) series. Pearson correlation coefficients were calculated by 40-year moving windows lagged backward by 5 years and range from red (positive correlation) to blue (negative correlation). Gray color indicates no data. Asterisks in **(A,B)** panels indicate *p*-value <0.1 (*), <0.05 (*), and <0.01 (***). Vertical red dashed lines indicate dates of construction of the first and second Monteith dam.

Investigation of the temporal stability of these associations also suggested that radial growth at none of the sites was significantly and consistently correlated with mean spring discharge or precipitation (Figure 6). After the 1970s, each mean site chronology, however, demonstrated a change from negative to positive correlations (*p* > 0.05) between the radial growth and mean spring discharge (Figure 6). The correlation between radial growth and average spring precipitation was mostly negative, with a trend toward positive correlations since about the 1990s at three of five sites (Figure 6). Overall, this weak evidence of a dam effect on riparian tree growth demonstrated the advantages of flood-rings over ring-width records in flood history studies when ring-porous species are available. It is also possible that the change created by the Monteith Dam was not strong enough to affect radial tree growth but still affected the earlywood vessels. In this case, further study on a larger number of rivers and hydrological contexts is needed to determine if ring widths can be used to supplement flood rings. However, little to no consistent responses between total and sub-annual tree-ring width and spring flood discharge has neither been found in riparian *Q. macrocarpa* (St. George et al., 2002; St. George and Nielsen, 2003) nor in *F. nigra* (Tardif and Bergeron, 1993; Tardif et al., 1997b; Kames et al., 2016; Nolin et al., 2021a; Tardif et al., 2021b). The inversion of correlation sign found between ring width and discharge after damming is consistent with previous studies of North American rivers (Reily and Johnson, 1982; Stromberg and Patten, 1990; Salzer et al., 1996; Coble and Kolb, 2012; Netsvetov et al., 2019). Although in our study, the change in the sign was the opposite, from a negative correlation before 1970 to a positive correlation after 1970. The weak and poorly significant correlations in the case of the Driftwood River also contrast with the strong and significant changes found in these studies. Most of these studies have, however, been conducted in arid to semiarid environments where water (precipitation and/or discharge) constitutes a limiting factor to growth, which is rarely the case near boreal rivers. Since the first dam was built about 100 years ago, it is also possible that the riparian forests of the Driftwood River are surviving forests within which the trees whose ring widths were severely affected may have died already and thus not appear in the data.

In conclusion, this study demonstrated the feasibility of extracting hydrological information from regulated rivers and in particular in the predam period. The flood rings successfully recorded the spring floods in the predam period, as indicated by comparison with instrumental and reconstructed records from nearby unregulated rivers. The number of trees recording flood rings, as well as their intensity within tree rings (F1, F2 types), decreased after the construction of the first dam in 1917–1918, and particularly after its replacement in 1952–1953. Compared to flood-ring chronologies, ring-width chronologies in the sampled trees were weakly correlated to the instrumental and reconstructed discharge of the Harricana River and did not record river regulation and at any distance to the shore. On the contrary, trees within 10 m from the shoreline recorded more well-defined flood rings and of higher intensity (F2) than those further away, thus presenting a better association with known regional flood years. Comparing to the downstream site to the dam, the upstream ones recorded significantly more flood rings in the postdam period, reemphasizing the importance of considering the position of the site along with the river continuum and site conditions in relation to flood exposure. Our results suggest that a sampling strategy taking advantage of tree sampling at a far distance upstream from the dam and along various hydrologic contexts may maximize the flood signal recorded by riparian ring-porous trees in regulated rivers. A good strategy might be to sample upstream of any natural or rapid restrictions in a river, as these restrictions back up some of the water to the riparian zone during flood events. Given the importance of hydropower in northern Canada and the need for hydrological proxies in the context of climate change, it remains critical to continue developing dendrohydrological research using riparian trees and to better understand the interaction between hydrological proxies and water-level regulation.

## Data Availability Statement

Relevant data for this study are available at Mendeley Data, v1. (http://dx.doi.org/10.17632/6pgc25nk27.1). All other data are available upon request to the corresponding author (alexandreflorent.nolin@uqat.ca or alexandreflorent.nolin@gmail.com).

## Author Contributions

AN: conceptualization, methodology, investigation, formal analysis, writing - original draft, writing - review and editing, and visualization. JT: conceptualization, methodology, investigation, writing - review and editing, supervision, project administration, and funding acquisition. FC: methodology, investigation, resources, data curation, and writing - review and editing. YB: conceptualization, writing - review and editing, supervision, project administration, and funding acquisition. All authors contributed to the article and approved the submitted version.

## Conflict of Interest

The authors declare that the research was conducted in the absence of any commercial or financial relationships that could be construed as a potential conflict of interest.

## Publisher’s Note

All claims expressed in this article are solely those of the authors and do not necessarily represent those of their affiliated organizations, or those of the publisher, the editors and the reviewers. Any product that may be evaluated in this article, or claim that may be made by its manufacturer, is not guaranteed or endorsed by the publisher.

## Footnotes

1
https://climexp.knmi.nl

2
http://www.thekingshighway.ca/list.htm
