# Supplementary material for: Flood-Rings Production Modulated by River Regulation in Eastern Boreal Canada
Source: Front Plant Sci. 2021 Oct 28;12:757280. doi: 10.3389/fpls.2021.757280 (PMC8581619; doi:10.3389/fpls.2021.757280)
Supplement: Supplementary file 1 [file Table_1.DOCX]

**Supplementary Materials**

**Table S1.** Change point analysis of mean site chronologies of tree-ring width. Results are given for each site (univariate analysis, n = 1 chronology) and each combination of mean site chronologies tested (multivariate analysis, n = 2, 3, 4 or 5 chronologies). Multivariate analyses were performed on the period common to the chronology or to the chronology combination used. Years were identified as significant change points when *p* < 0.05.

| **Site(s)** | **Common period** | **No. of change points** | **Years identified as significant (p < 0.05) change points** | | | | | | |
| --- | --- | --- | --- | --- | --- | --- | --- | --- | --- |
| RPR1 | 1930-2017 | 0 |  |  |  |  |  |  |  |
| DFR1 | 1917-2017 | 0 |  |  |  |  |  |  |  |
| DFR2 | 1879-2017 | 0 |  |  |  |  |  |  |  |
| MOL1 | 1898-2017 | 0 |  |  |  |  |  |  |  |
| MOL2 | 1852-2017 | 1 |  | 1914 |  |  |  |  |  |
| RPR1, DFR1, DFR1, MOL1, MOL2 | 1930-2017 |  |  |  |  |  | 1945 |  | 1975 |
| DFR1, DFR2, MOL1, MOL2 | 1917-2017 | 2 |  |  |  | 1941 |  | 1974 |  |
| DFR2, MOL1, MOL2 | 1879-2017 | 3 | 1909 |  |  | 1941 |  | 1974 |  |
| MOL1, MOL2 | 1898-2017 | 2 |  |  | 1917 |  |  | 1974 |  |
